# Right heart strain in arrhythmogenic right ventricular cardiomyopathy: implications for cardiovascular outcome

**DOI:** 10.1093/ehjci/jeae117

**Published:** 2024-04-29

**Authors:** Shehab Anwer, Lauren Stollenwerk, Neria E Winkler, Francesca Guastafierro, Monika Hebeisen, Deniz Akdis, Ardan M Saguner, Corinna Brunckhorst, Firat Duru, Felix C Tanner

**Affiliations:** Department of Cardiology, University Heart Center, University Hospital Zürich and University of Zürich, Raemistrasse 100, 8091 Zürich, Switzerland; Center for Translational and Experimental Cardiology (CTEC), University Hospital Zürich and University of Zürich, Wagistrasse 12, 8952 Schlieren, Zürich, Switzerland; Department of Cardiology, University Heart Center, University Hospital Zürich and University of Zürich, Raemistrasse 100, 8091 Zürich, Switzerland; Department of Cardiology, Bern University Hospital, Bern, Switzerland; Department of Cardiology, University Heart Center, University Hospital Zürich and University of Zürich, Raemistrasse 100, 8091 Zürich, Switzerland; Center for Translational and Experimental Cardiology (CTEC), University Hospital Zürich and University of Zürich, Wagistrasse 12, 8952 Schlieren, Zürich, Switzerland; Humanitas Research Hospital, Istituto di Ricovero e Cura a Carattere Scientifico, Milan, Italy; Department of Cardiology, University Heart Center, University Hospital Zürich and University of Zürich, Raemistrasse 100, 8091 Zürich, Switzerland; Department of Biostatistics, Epidemiology, Biostatistics and Prevention Institute, University of Zurich, Zurich, Switzerland; Department of Cardiology, University Heart Center, University Hospital Zürich and University of Zürich, Raemistrasse 100, 8091 Zürich, Switzerland; Center for Translational and Experimental Cardiology (CTEC), University Hospital Zürich and University of Zürich, Wagistrasse 12, 8952 Schlieren, Zürich, Switzerland; Division of Cardiology, GZO Zurich Regional Health Center Wetzikon, Wetzikon, Switzerland; Department of Cardiology, University Heart Center, University Hospital Zürich and University of Zürich, Raemistrasse 100, 8091 Zürich, Switzerland; Center for Translational and Experimental Cardiology (CTEC), University Hospital Zürich and University of Zürich, Wagistrasse 12, 8952 Schlieren, Zürich, Switzerland; Department of Cardiology, University Heart Center, University Hospital Zürich and University of Zürich, Raemistrasse 100, 8091 Zürich, Switzerland; Center for Translational and Experimental Cardiology (CTEC), University Hospital Zürich and University of Zürich, Wagistrasse 12, 8952 Schlieren, Zürich, Switzerland; Department of Cardiology, University Heart Center, University Hospital Zürich and University of Zürich, Raemistrasse 100, 8091 Zürich, Switzerland; Center for Translational and Experimental Cardiology (CTEC), University Hospital Zürich and University of Zürich, Wagistrasse 12, 8952 Schlieren, Zürich, Switzerland; Department of Cardiology, University Heart Center, University Hospital Zürich and University of Zürich, Raemistrasse 100, 8091 Zürich, Switzerland; Center for Translational and Experimental Cardiology (CTEC), University Hospital Zürich and University of Zürich, Wagistrasse 12, 8952 Schlieren, Zürich, Switzerland

**Keywords:** echocardiography, speckle-tracking echocardiography, strain imaging, right atrium, right ventricle, arrhythmogenic right ventricular cardiomyopathy, ARVC

## Abstract

**Aims:**

Arrhythmogenic right ventricular cardiomyopathy (ARVC) is characterized by progressive myocardial dysfunction and associated with an increased risk of major cardiovascular (CV) events. To determine right heart strain (ventricular and atrial global longitudinal strain (RVGLS and RAGLS) in patients with definite ARVC and its association with adverse events during follow-up.

**Methods and results:**

RVGLS and RAGLS were analysed in focused right heart apical views from 70 patients using TomTec ImageArena and association with a composite endpoint was determined (sustained ventricular arrhythmia and cardiovascular death). Over a median follow-up duration of 4.9 years, 26 (37%) patients met the endpoint. RVGLS was significantly impaired in the event group (−11.5 [−13.3 to −10.2] %) vs. the no-event group (−15.8 [−17.1 to −14.5] %, *P* < 0.001), and so was RAGLS (22.8 [21.4–27.4] % vs. 31.5 [25.1–39.6] %, respectively, *P* < 0.001). In Cox regression, RVGLS (HR 1.36, *P* < 0.001) and RAGLS (HR 0.92, *P* = 0.002) were associated with a higher risk of adverse events. In multivariable Cox regression models, RVGLS and RAGLS remained independent of and were incremental to age, gender, and conventional RV parameters, and model fitness was improved when RVGLS and RAGLS were applied together rather than alone.

**Conclusion:**

RVGLS and RAGLS are more impaired in patients with adverse events and associated with adverse events independent of age, gender, and conventional RV parameters. When RVGLS and RAGLS are applied together, prediction models are improved suggesting that right heart strain may form part of the echocardiographic routine protocol in patients with ARVC.

## Graphical Abstract

**Graphical Abstract:**
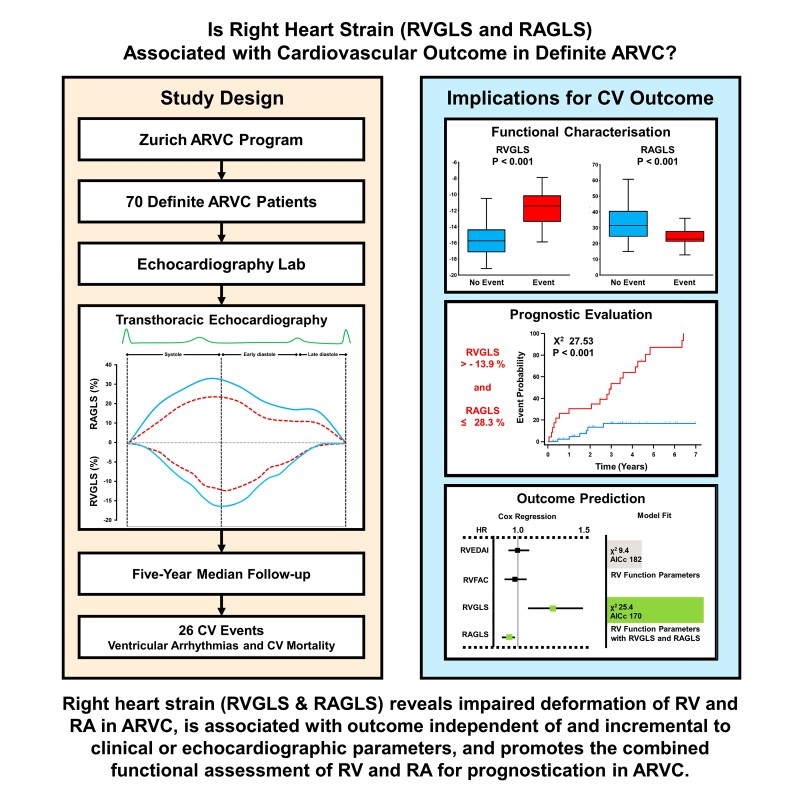

**See the editorial comment for this article ‘Looking beyond the right ventricular strain: right atrial strain in arrhythmogenic right ventricular cardiomyopathy', by J. Ambrožič and M. Cvijić, https://doi.org/10.1093/ehjci/jeae119.**

## Introduction

Arrhythmogenic right ventricular cardiomyopathy (ARVC) typically manifests in young adulthood, affects men more often than women, and predisposes to ventricular arrhythmia and sudden cardiac death (SCD). Initially, ARVC was thought to affect the right ventricle (RV), while biventricular and isolated left ventricular (LV) forms were recognized in later studies.^1–6^ The gene mutations linked to ARVC primarily induce abnormalities of intercellular junctions such as desmosomes that causes the detachment and degeneration of cardiomyocytes leading to fibro-fatty transformation with scarring of the myocardium. Such alterations eventually impact the structure and function of the ventricles as well as the atria of affected hearts.^1^

Due to its widespread availability and multifaceted application, echocardiography is an integral component of the clinical work-up in ARVC including diagnosis, follow-up, and prognostic evaluation. Recent methodological advances such as speckle-tracking echocardiography (STE) allow to determine the deformation of all the cardiac cavities, exhibit very good reproducibility, and are particularly useful for assessing outcome associations. In this context, STE-derived strain analysis may be more appropriate than conventional echocardiographic parameters as it may detect early regional or global mechanical alterations before overt myocardial dysfunction becomes manifest.^6–10^ Current evidence advocates strain analysis for characterizing cardiac function and evaluating cardiovascular outcome in ARVC, specifically RV global longitudinal strain (RVGLS),^10–18^ and, more recently, right atrial (RA) global longitudinal strain (RAGLS).^3^ While the potential of RAGLS for phenotype characterization and outcome assessment in ARVC has been recognized recently, its usefulness compared with RVGLS is not known, and the combined analysis of RVGLS and RAGLS has not been reported yet.

This study aimed at exploring (i) the association of RVGLS and RAGLS with outcome in ARVC, and (ii) whether their combined analysis improves outcome prediction.

## Methods

### Study design

Patients from the prospective Swiss ARVC Registry (www.arvc.ch) were studied in this analysis. Inclusion criteria consisted of: (i) diagnosis of definite ARVC according to the modified 2010 Task Force Criteria,^14^ (ii) age 16 years or older at inclusion date, (iii) follow-up duration of at least 1 year after inclusion, (iv) availability of a comprehensive echocardiographic examination allowing conventional and strain analysis according to current recommendations.^3,15–25^ Seventy patients fulfilled the criteria and were included in the study. Eight patients did not fulfil the criteria for the following reasons: one patient was <16 years old, one patient had a follow-up duration of <1 year, two patients were lost to follow-up, and four patients had no digitized echo available. All patients gave their written informed consent. The study was approved by the authorized ethics committee (Kantonale Ethikkommission Zürich).

### Transthoracic echocardiography

Transthoracic echocardiography was performed between January 2009 and December 2022 using commercially available equipment: iE33 or Epiq 7, Philips Medical Systems, The Netherlands; Vivid7, E9 or E95, GE Healthcare, USA.^3^ All echocardiographic examinations were carried out by professional investigators according to current recommendations.^15–20^

TomTec Image Arena (TTA2 Build 468168) Cardiac Performance Analysis (V1.4.0.109, Philips Medical Systems, The Netherlands) was used for offline measurement of RV and RA deformation by two-dimensional STE as this software is compatible with equipment from different vendors and was applied to define normal strain values for all the cardiac chambers.^17–25^ RVGLS and RAGLS were measured in RV focused apical four-chamber view according to current recommendations.^15–25^ The Endocardial border was traced manually using dedicated ventricular and atrial strain modules excluding any trabeculations and avoiding the pericardium. End-diastole was defined using M-mode as the last frame before tricuspid valve closure and end-systole as the last frame before tricuspid valve opening. RVGLS was calculated by averaging RV peak systolic strain based on a six-segment model and RV free wall strain (RVFWS) by the three segments of the RV free wall. RAGLS represents the reservoir phase of RA deformation and is defined as the difference between the nadir of the RA strain curve and the peak of the strain curve.^15–25^

Strain analysis of 15 (20%) echocardiographic examinations was performed by two observers to investigate interobserver variability and repeated by the main observer after three months to determine intra-observer variability using the same software version for all the measurements. Reproducibility (interobserver variability) and repeatability (intra-observer variability) were examined by an intra-class correlation coefficient test.

### Follow-up and cardiovascular events

The first echocardiographic examination allowing target strain analysis marked the date of study inclusion. Follow-up time was defined from that echocardiographic exam to the first occurrence of a cardiovascular event.

A combined endpoint was defined as the study endpoint and included sustained ventricular tachycardia, ventricular fibrillation, appropriate implantable cardioverter-defibrillator (ICD) discharge, cardiopulmonary resuscitation, SCD, heart transplantation, and/or cardiovascular mortality. To simplify reading, this combined endpoint is referred to as a ‘cardiovascular event’ or ‘event’. Arrhythmias were registered by ECG, Holter ECG, or cardiac device monitoring. Patient reports or phone calls were used for the remaining endpoints.

### Statistics

Statistical analysis was performed using MedCalc for Windows (Version 18.2.1., MedCalc Software, Ostend, Belgium), RStudio (v2023.12) with R (v4.3.0) running standard statistical package (Posit, USA).^3^ Shapiro–Wilk test was used for testing the normality of distribution, with deviation from normal distribution reported in most of the data. Continuous variables were presented by their median and interquartile range [IQR] and compared using Mann–Whitney *U* test.^3^ Categorical variables were described as numbers and percentages, and compared using Fischer’s exact test.^3^ Time-dependent associations with the study endpoint (CV Event) were tested in uni- and multivariable Cox regression models. Proportional hazard assumptions were assessed for all the models using the scaled Schoenfeld residuals, with all variables meeting the assumption. Cox regression analysis of variance and the corrected Akaike information criterion (AICc) were used to test model fit. Receiver observer characteristics analysis determined the sensitivity and specificity of reported cut-offs and were indicated as Youden’s Index (Youden), area under the curve (AUC), and its 95% confidence interval. The best cut-off values to dichotomize the study population were derived from ROC analyses. Event probability was determined with Kaplan–Meier curves according to the best cut-off values as well as to study population tertiles. *P*-values < 0.05 were considered statistically significant.

## Results

### Baseline clinical characteristics

A total of 70 patients with definite ARVC were included (*Table 1*). Their median age was 50.0 [32.3–58.6] years. The majority consisted of men (48, 69%) and corresponded to index patients (68, 97%). Diagnosis of ARVC happened within a median of 294 [186–450] days before study inclusion. An ICD had been implanted before study inclusion in 30 patients (43%).

**Table 1:** Baseline clinical characteristics, median [IQR]

Parameters	Overall (*n* = 70)
Age, years	50.0 [32.3–58.6]
Men, *n* (%)	48 (69)
BSA, m^2^	1.9 [1.8–2.1]
Index patients, *n* (%)	68 (97)
Family members, *n* (%)	2 (3)
TFC 2010, major criteria	2 [1–2]
TFC 2010, minor criteria	2 [2–3]
Positive ARVC genotype, *n* (%)	48 (69)
History of VT, *n* (%)	13 (18)
RV segments with RWMA, *n* (%)	4 [3–5]
Epsilon wave, *n* (%)	7 (10)
T-wave inversion, *n* (%)	58 (83)
NYHA III/IV, *n* (%)	9 (13)
ICD before inclusion	30 (43)
Time diagnosis to inclusion, days	294 [186–450]
Follow-up period, days	1804 [1353–2680]

BSA, body surface area; TFC 2010, Task Force Criteria; ARVC, arrhythmogenic right ventricular cardiomyopathy; VT, ventricular tachycardia; RWMA, regional wall motion abnormalities; NYHA, New York Heart Association; ICD, implantable cardioverter-defibrillator.

### Follow-up and cardiovascular events

The patients were followed-up for a median of 1804 [1353–2680] days. During follow-up, 26 (37%) patients experienced a cardiovascular event and were included in the event group, while the remaining patients were included in the no-event group (44, 63%).

### Conventional echocardiography

The patient population showed structural and functional impairment of both RV and RA as detailed in *Table 2*. Among the patients with event, some right heart parameters exhibited more pronounced impairment compared with those without. In those with event, right ventricular end-diastolic area index (RVEDAI) was larger (17.5 [16.0–22.9] cm^2^/m^2^) than in those without (16.5 [14.1–19.2] cm^2^/m^2^, *P* = 0.017) and right ventricular fractional area change (RVFAC) was lower (22.8 [18.9–26.8] %) than in those without (28.7 [21.4–32.3] %, *P* = 0.011), while tricuspid annular plane systolic excursion (TAPSE) and tricuspid regurgitation severity did not differ significantly between the groups. In those with event, right atrial volume index (RAVI) was larger (39.0 [27.0–64.0] mL/m^2^) than in those without (29.0 [25.0–40.0] mL/m^2^, *P* = 0.029), while RA major and minor diameters did not differ significantly between the groups.

**Table 2:** Baseline echocardiographic characteristics [median, IQR]

Parameters	Overall (*n* = 70)	No event (*n* = 44)	Event (*n* = 26)	*P*
RVEDAI, cm^2^/m^2^	16.9 [14.8–19.6]	16.5 [14.1–19.2]	17.5 [16.0–22.9]	0.017*
TAPSE, mm	19 [15–22]	19 [17–23]	18 [14–21]	0.142
RVFAC, %	25.2 [21.2–30.2]	28.7 [21.4–32.3]	22.8 [18.9–26.8]	0.011*
RVGLS, %	−14.4 [−16.5 to −12.5]	−15.8 [−17.1 to −14.5]	−11.5 [−13.3 to −10.2]	<0.001*
RVFWS, %	−14.1 [−21.2 to −6.9]	−16.6 [−21.7 to −10.8]	−11.2 [−17.7 to −4.2]	<0.001*
RA^major^, mm/m^2^	26.0 [23.0–32.0]	26.5 [23.0–31.5]	26.0 [23.0–34.0]	0.526
RA^minor^, mm/m^2^	23.0 [22.0–26.0]	23.0 [21.0–25.5]	25.0 [22.0–30.0]	0.103
RAVI mL/m^2^	35.0 [25.0–45.0]	29.0 [25.0–40.0]	39.0 [27.0–64.0]	0.029*
RAGLS, %	27.7 [22.7–33.1]	31.5 [25.1–39.6]	22.8 [21.4–27.4]	<0.001*
LVEF, %	55 [50–61]	54.5 [50.0–60.5]	57.0 [46.0–62.0]	0.711
TR III/IV, *n* (%)	5 (7)	1 (1)	4 (6)	0.060

RVEDAI, right ventricular end-diastolic area index; TAPSE, tricuspid annular plane systolic excursion; RVFAC, right ventricular fractional area change; RVGLS, right ventricular global longitudinal strain; RVFWS, right ventricular free wall strain; RA, right atrium; RA^major^, right atrial major dimension; RA^minor^, right atrial minor dimension; RAVI, right atrial volume index; RAGLS, right atrial global longitudinal strain; LVEF, left ventricular ejection fraction; TR III/IV, tricuspid regurgitation grade III/IV.

^*^Significant *P*-value (< 0.05).

### Right heart strain

A strong inter- and intra-observer agreement was found when intra-class correlations were studied between measurements for RVGLS (*r* = 0.90 and 0.91, respectively, *P* < 0.001) and RAGLS (*r* = 0.90 and 0.90, respectively, *P* < 0.001).

**Figure 1:**
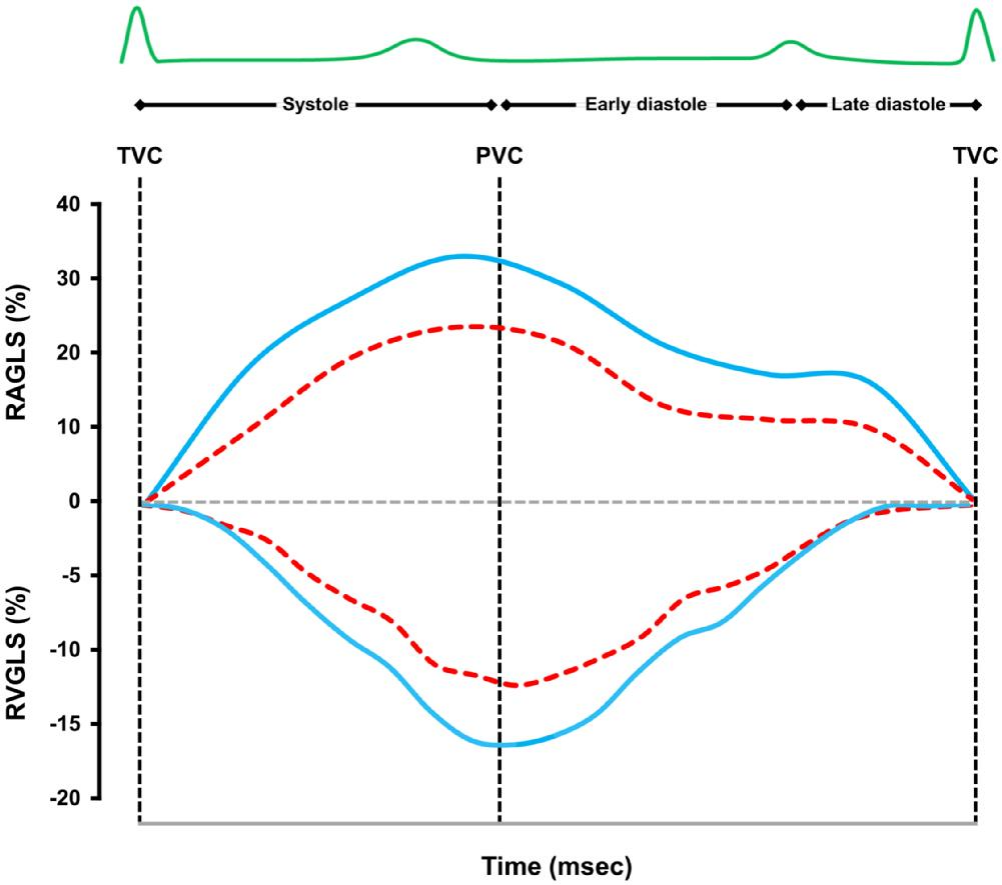
Strain curves of right ventricle (RVGLS) and right atrium (RAGLS) of ARVC patients. Impairment of RVGLS (lower section) and RAGLS (upper section) values was reported among ARVC patients who experienced cardiovascular event (dashed curve) compared with those who did not (solid curve).

**Figure 2:**
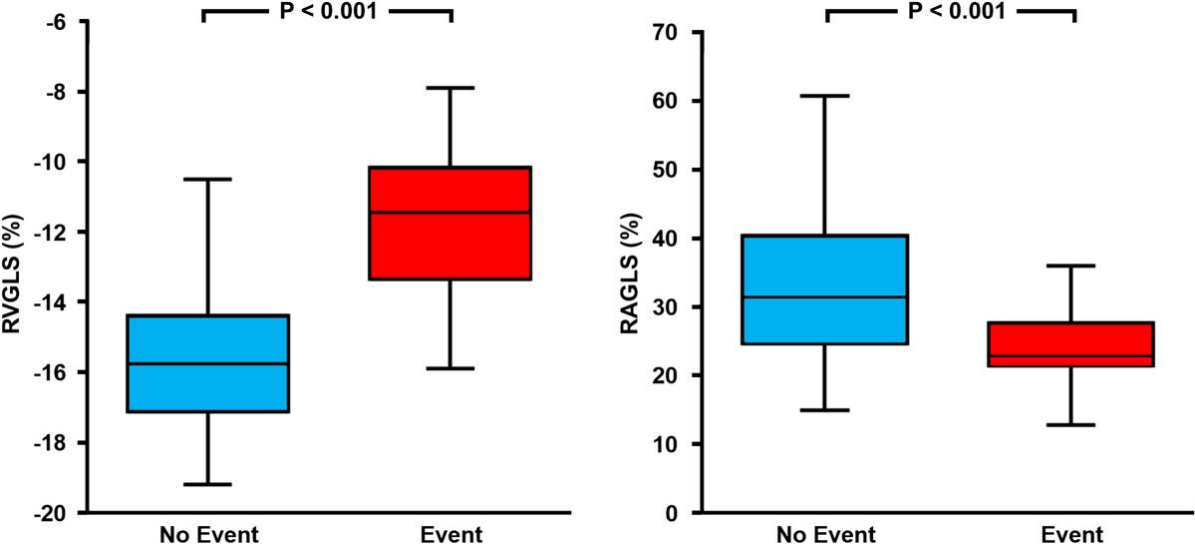
Strain values of right ventricle (RVGLS) and right atrium (RAGLS) in ARVC patients. RVGLS (left panel) and RAGLS (right panel) were impaired among those with events compared with those without event.

RVGLS (*Figures 1* and *2*, *Table 2*) was significantly impaired in patients with event (−11.5 [−13.3 to −10.2] %) compared with those without (−15.8 [−17.1 to −14.5] %, *P* < 0.001). Similarly, RVFWS (*Table 2*) was significantly impaired in patients with event (−11.2 [−17.7 to −4.2] %) compared with those without (*P* < 0.001). RAGLS (*Figures 1* and *2*, *Table 2*) was significantly impaired in patients with event (22.8 [21.4–27.4] %) compared with those without (31.5 [25.1–39.6] %, *P* < 0.001).

**Figure 3:**
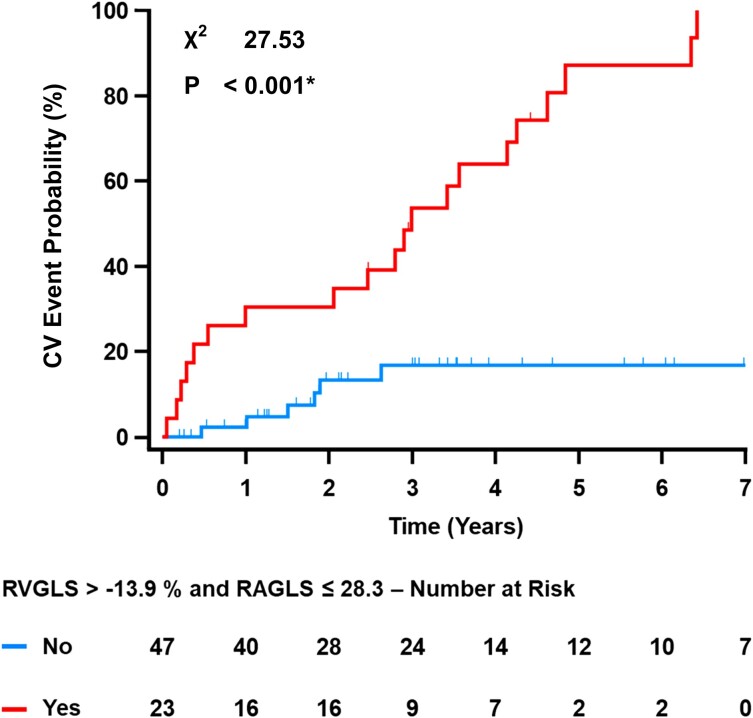
Right heart strain and cardiovascular (CV) event probability in ARVC patients. ARVC patients with both right ventricular strain (RVGLS) > −13.9% and right atrial strain (RAGLS) ≤ 28.3% exhibited a higher probability of events than those with both RVGLS ≤ −13.9% and RAGLS > 28.3%.

According to ROC analyses, RVGLS > −13.9% (Youden = 0.72, AUC 0.92 [0.83–0.97]) and RAGLS ≤ 28.3% (Youden = 0.52, AUC 0.77 [0.83–0.97]) represented the best cut-off values for differentiating patients at higher risk of an event. When the cut-off values were used to dichotomize the study population in Kaplan–Meier analyses, patients with RVGLS > −13.9% or RAGLS ≤ 28.3% exhibited a higher event probability during follow-up (*P* < 0.001 and *P* = 0.001, respectively; Supplementary data online, *Figure S1*). With RVFWS instead of RVGLS, similar results were obtained (data not shown). When the cut-off values for RVGLS and RAGLS were applied together, patients fulfilling both criteria had a significantly higher probability of an event during follow-up (*P* < 0.001, *Figure 3*).

The Kaplan–Meier analyses were also performed with tertiles of RVGLS or RAGLS, respectively. The tertiles allowed a good evaluation of event probability with a higher event risk in those with impaired strain (RVGLS: *P* < 0.0001; RAGLS: *P* = 0.0006; Supplementary data online, *Figure S2*).

### Association with outcome

There was a significant association of RVEDAI, RVFAC, RVGLS, and RAGLS with a higher risk of an event (*Table 3*), while other models such as age, gender, TAPSE, or tricuspid regurgitation were not associated (*Table 3*).

**Table 3:** Univariable Cox regression analysis

Variables	Cox regression		
	HR	95% CI	*P*
Age, year	0.99	0.97–1.02	0.662
Gender, men	1.62	0.65–4.04	0.299
RVEDAI, cm^2^/m^2^	1.07	1.01–1.13	0.016*
TAPSE, mm	0.93	0.86–1.00	0.056
RVFAC, %	0.91	0.85–0.98	0.009*
RVGLS, %	1.36	1.18–1.58	<0.001*
RVFWS, %	1.20	1.08–1.47	0.001*
RAVI, mL/m^2^	1.01	1.00–1.02	0.020*
RAGLS, %	0.92	0.88–0.97	0.002*
TR III/IV	1.94	0.66–5.70	0.226

HR, hazard ratio; 95% CI, 95% confidence interval of the hazard ratio; *P*, *P*-value; χ^2^, chi-square statistic; AICc, Akaike Information Criterion corrected for small sample size; RVEDAI, right ventricular end-diastolic area index; TAPSE, tricuspid annular plane systolic excursion; RVFAC, right ventricular fractional area change; RVGLS, right ventricular global longitudinal strain; RVFWS, right ventricular free wall strain; RAVI, right atrial volume index; RAGLS, right atrial global longitudinal strain; TR III/IV, tricuspid regurgitation grade III/IV.

^*^Significant *P*-value (< 0.05).

A multivariable Cox regression model demonstrated that outcome association of RVGLS or RAGLS was independent of age and gender (clinical model, *Table 4*). When RVGLS and RAGLS were evaluated in the model together, independence of both parameters from age and gender was maintained and model fit improved (*Table 4*). Other multivariable models revealed that outcome association of RVGLS or RAGLS was independent of RVEDAI and RVFAC (*Table 5*) or independent of RAVI, respectively (*Table 6*). Again, independence of RVGLS and RAGLS was maintained and model fit improved when they were examined in the models together (*Tables 5* and *6*).

**Table 4:** Multivariable Cox regression—clinical model

Variables	Cox regression			Model fit		
	HR	95% CI	*P*	χ^2^	*P*	AICc
Age, year	0.99	0.97–1.02	0.517	1.58	0.455	189
Gender, men	1.71	0.68–4.31	0.259			
Age, year	0.99	0.96–1.02	0.588	19.35	<0.001*	174
Gender, men	1.75	0.59–5.20	0.312			
RVGLS, %	1.36	1.17–1.59	<0.001*			
Age, year	0.98	0.96–1.01	0.176	14.85	0.002*	178
Gender, men	1.28	0.49–3.32	0.612			
RAGLS, %	0.92	0.87–0.97	0.002*			
Age, year	0.98	0.96–1.01	0.303	26.34	<0.001*	169
Gender, men	1.63	0.55–4.87	0.382			
RVGLS, %	1.28	1.10–1.50	0.001*			
RAGLS, %	0.93	0.87–0.99	0.015*			

HR, hazard ratio; 95% CI, 95% confidence interval of the hazard ratio; *P*, *P*-value; χ^2^, chi-square statistic; AICc, Akaike Information Criterion corrected for small sample size; RVGLS, right ventricular global longitudinal strain; RAGLS, right atrial global longitudinal strain.

^*^Significant *P*-value (< 0.05).

**Table 5:** Multivariable Cox regression—right ventricle model

Variables	Cox regression			Model fit		
	HR	95% CI	*P*	χ^2^	*P*	AICc
RVEDAI, cm^2^/m^2^	1.04	0.98–1.11	0.206	9.38	0.009	182
RVFAC, %	0.93	0.86–1.00	0.038*			
RVEDAI, cm^2^/m^2^	1.01	0.94–1.09	0.812	20.67	<0.001*	172
RVFAC, %	0.95	0.88–1.02	0.156			
RVGLS, %	1.30	1.10–1.53	0.002*			
RVEDAI, cm^2^/m^2^	1.03	0.97–1.10	0.329	16.18	0.001*	177
RVFAC, %	0.95	0.88–1.03	0.250			
RAGLS, %	0.94	0.89–0.99	0.019*			
RVEDAI, cm^2^/m^2^	1.00	0.93–1.09	0.855	25.40	<0.001*	170
RVFAC, %	0.98	0.90–1.07	0.614			
RVGLS, %	1.27	1.08–1.50	0.004*			
RAGLS, %	0.94	0.88–1.00	0.050			

HR, hazard ratio; 95% CI, 95% confidence interval of the hazard ratio; *P*, *P*-value; χ^2^, chi-square statistic; AICc, Akaike Information Criterion corrected for small sample size; RVEDAI, right ventricular end-diastolic area index; RVFAC, right ventricular fractional area change; RVGLS, right ventricular global longitudinal strain; RAGLS, right atrial global longitudinal strain.

^*^Significant *P*-value (< 0.05).

**Table 6:** Multivariable Cox regression—right atrium model

Variables	Cox regression			Model fit		
	HR	95% CI	*P*	χ^2^	*P*	AICc
RAVI, mL/m^2^	1.00	0.99–1.01	0.459	13.54	0.001*	177
RAGLS, %	0.93	0.88–0.98	0.007*			
RAVI, mL/m^2^	1.00	0.99–1.01	0.795	18.31	< 0.001*	173
RVGLS, %	1.35	1.15–1.58	< 0.001*			
RAVI, mL/m^2^	1.00	0.99–1.01	0.620	25.34	< 0.001*	168
RAGLS, %	0.93	0.87–0.99	0.017*			
RVGLS, %	1.31	1.12–1.54	0.001*			

HR, hazard ratio; 95% CI, 95% confidence interval of the hazard ratio; *P*, *P*-value; χ^2^, chi-square statistic; AICc, Akaike Information Criterion corrected for small sample size; RAVI, right atrial volume index; RVGLS, right ventricular global longitudinal strain; RAGLS, right atrial global longitudinal strain.

^*^Significant *P*-value (< 0.05).

## Discussion

This study assesses right heart strain in patients with definitive ARVC and demonstrates that RVGLS and RAGLS are more impaired in patients with events, RVGLS and RAGLS differentiate between patients with and without events, RVGLS and RAGLS are associated with events independent of clinical or echocardiographic parameters, and RVGLS and RAGLS allow improved event prediction when applied together. These observations highlight the importance of a comprehensive echocardiographic right heart assessment including RVGLS and RAGLS in ARVC patients.

Conventional echocardiography provides information on cardiac dimensions and function. The latter is based on changes in size of cardiac structures or displacement of defined landmarks. Hence, FAC describes RV area shortening during systole in apical four-chamber view, and TAPSE determines longitudinal displacement of the tricuspid annulus during systole in the same view. In contrast, strain imaging provides information beyond the conventional parameters because it determines myocardial deformation during the cardiac cycle and on a segmental level. Furthermore, strain imaging can detect even subtle mechanical alterations of all the cardiac chambers including the atria. Consistent with this concept, strain imaging was found to be more sensitive than conventional parameters and of particular importance for outcome assessment by echocardiography.^15–20^

Strain imaging analyses the myocardium itself that may account for its improved event differentiation and superior outcome prediction.^15–20^ The current study confirms that strain imaging generates a better understanding of cardiac function than conventional echocardiographic parameters both at the ventricular and atrial level.^3,8,15–25^ This is underscored by the observation that outcome association of RVGLS and RAGLS remains independent of conventional echocardiographic parameters as well as dominant clinical factors such as age or gender. The strong and consistent alterations in RAGLS indicate that the RA is affected by the pathological alterations occurring in ARVC and that the RA may be considered a useful indicator of phenotypic manifestations during disease progression. Thus, it seems reasonable to integrate deformation imaging of both RV and RA in the routine echocardiographic assessment of ARVC patients.^23–28^

The current study is the first to describe echocardiographic deformation of the right heart with RVGLS and RAGLS derived from a single dedicated modified apical view. Furthermore, the study explores the association of RVGLS and RAGLS as well as their combination with cardiovascular outcome. The findings demonstrate that addition of RAGLS to the more often measured RVGLS may not only promote understanding of the cardiac phenotype but also improve event prediction by outcome association. Hence, the combined assessment of RV and RA strain in this context seems to be of additional benefit and may have incremental value in the echocardiographic work-up of patients with ARVC.^17–20,23–28^

Several observations are supporting the interpretation that ARVC does not only affect the ventricles but also the atria.^25–34^ First of all, the genetic background of ARVC is consistent with the involvement of cardiomyocytes in both ventricles and atria.^25–31^ In addition, secondary affection of the RA through atrioventricular coupling would be expected with disease progression even if initial phenotypic manifestations were assumed to be restricted to the RV. In fact, the RA was found to be altered in ARVC patients at the histopathological, structural, and functional level.^25–34^ Hence, the genetic and functional changes occurring in ARVC eventually lead to fibrosis of the right heart involving primarily the RV but also the RA. Fibrosis is known to negatively affect all the events involved in regulating atrial reservoir function, namely systolic movement of the ventricular base towards the apex, atrial filling pressure, and atrial contractility. Therefore, it is conceivable that RAGLS, which measures atrial reservoir function, is an imaging tool with a large potential for phenotype characterization and outcome prediction in cardiomyopathies such as ARVC. Right heart strain has the potential to improve risk stratification in ARVC patients like recent studies adding programmed ventricular stimulation or RV free wall strain and RV regional deformation patterns to the ARVC risk calculator.^10,28,31–34^

The application of RAGLS in clinical practice seems quite straightforward. Acquisition of RVGLS and RAGLS can be attained in a single dedicated modified apical view. Based on the dedicated RV view, the only adaptation required for optimal RAGLS may consist of increasing the line-density of the sector in order to obtain optimal conditions in the far field. Since this represents little additional effort on the one hand and may offer major advantages for phenotype characterization and outcome assessment on the other hand, it seems well justified to acquire an echocardiographic view optimized for RAGLS in a routine examination.

### Limitations

The single centre retrospective design as well as the moderate number of patients and events limits this study. Since standardized strain measurements between different vendors are currently not available, reproducibility across vendors might be limited in a routine clinical setting unless the TomTec Image Arena software is used for strain analysis.^3,29^

## Conclusions

In definitive ARVC, RVGLS and RAGLS are more impaired in patients with events, differentiate between those with and without events, and are associated with events independent of clinical or conventional echocardiographic parameters. When RVGLS and RAGLS are applied together, prediction models are improved. This suggests that both RVGLS and RAGLS may form part of the echocardiographic routine protocol in ARVC.

## Supplementary data

Supplementary data are available at *European Heart Journal - Cardiovascular Imaging* online.

## Supplementary Material

jeae117_Supplementary_Data

## Data Availability

The data that support the findings of this study are available upon reasonable request from the authors.
