# Supplementary material for: Right heart strain in arrhythmogenic right ventricular cardiomyopathy: implications for cardiovascular outcome
Source: Eur Heart J Cardiovasc Imaging. 2024 Apr 29;25(8):1061–8. doi: 10.1093/ehjci/jeae117 (PMC11288757; doi:10.1093/ehjci/jeae117)
Supplement: jeae117_Supplementary_Data [file jeae117_supplementary_data.zip › RHS ARVC and CV Outcome - Supplementary Material EHJCVI.pdf]

Right Heart Strain In Arrhythmogenic Right Ventricular Cardiomyopathy: Implications For Cardiovascular Outcome

Shehab Anwer, Lauren Stollenwerk, Neria E. Winkler, Francesca Guastafierro, Monika Hebeisen, Deniz Akdis, Ardan M. Saguner, Corinna Brunckhorst, Firat Duru, Felix C. Tanner

European Heart Journal - Cardiovascular Imaging, 2024; jeae117, https://doi.org/10.1093/ehjci/jeae117

Supplementary Materials

Thank you for reading our work!

Supplementary Figure 1

Event probability according to the ROC cut-off of right ventricle (RVGLS; left panel) or right atrium (RAGLS; right panel).

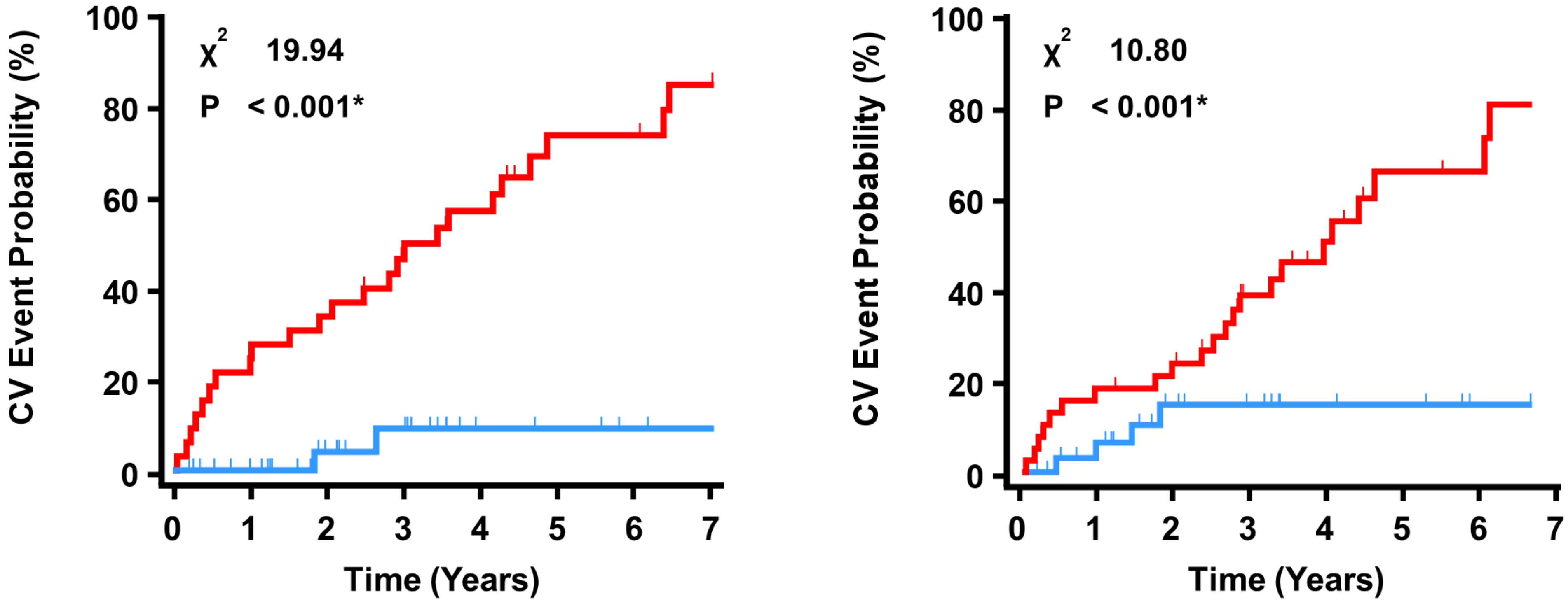

Number at Risk

	0	1	2	3	4	5	6	7
RVGLS ≤ -13.9%	33	25	22	15	12	6	6	2
RVGLS > -13.9%	37	31	22	18	9	8	6	5

Number at Risk

	0	1	2	3	4	5	6	7
RAGLS > 28.3%	33	27	17	15	10	9	8	5
RAGLS ≤ 28.3%	37	29	27	18	11	5	4	2

Thank you for reading our work!

Supplementary Figure 2

Event probability according to strain tertiles of right ventricle (RVGLS; left panel) or right atrium (RAGLS; right panel).

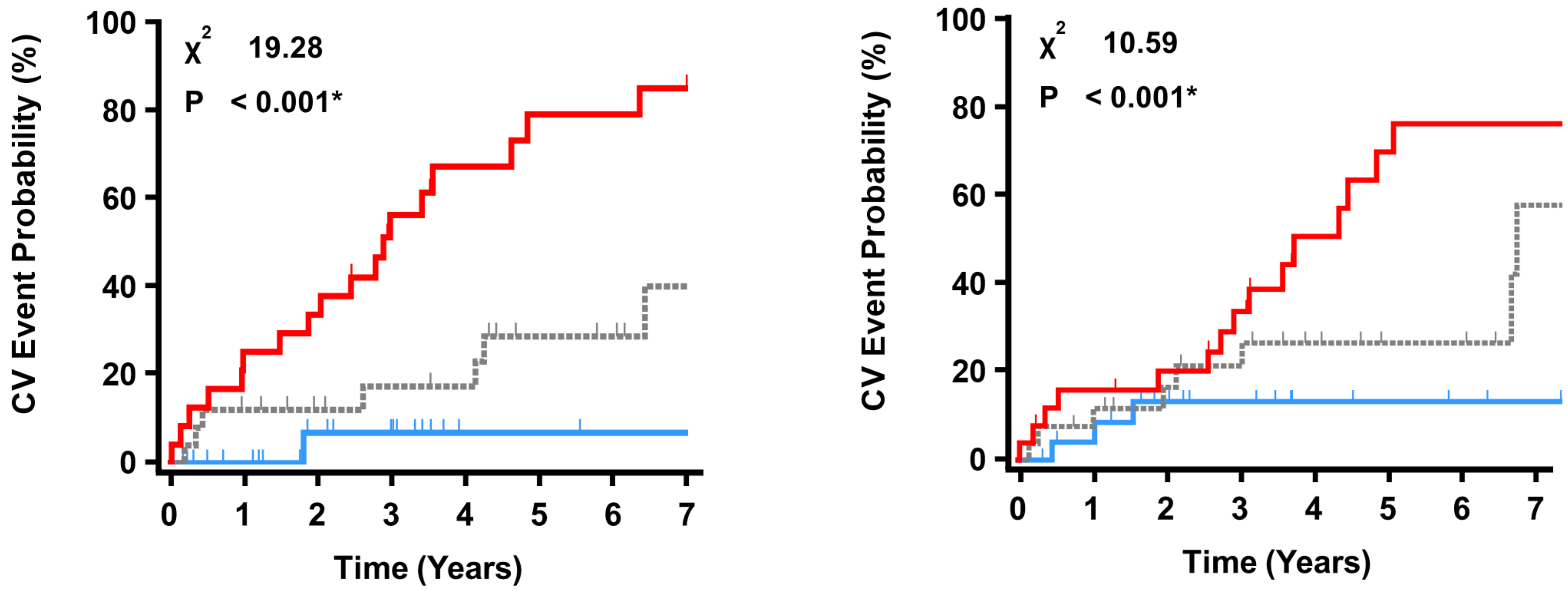

Number at Risk

**RVGLS 1st Tertile[-19.1 – -15.9]%**							
23	18	12	10	2	2	1	1
**RVGLS 2nd Tertile [-15.7 – -13.1]%**							
24	20	17	15	14	9	8	5
**RVGLS 3rd Tertile [-12.7 – -7.9]%**							
23	18	15	8	5	3	3	1

Number at Risk

**RAGLS 1st Tertile [32.2 – 60.7]%**							
23	19	13	11	7	6	5	3
**RAGLS 2nd Tertile [23.7 – 31.5]%**							
24	19	15	12	8	6	5	2
**RAGLS 3rd Tertile [10.7 – 23.1]%**							
23	18	16	10	6	2	2	2

Thank you for reading our work!
