# Supplementary figures and images for: Right heart strain in arrhythmogenic right ventricular cardiomyopathy: implications for cardiovascular outcome
Source: Eur Heart J Cardiovasc Imaging. 2024 Apr 29;25(8):1061–8. doi: 10.1093/ehjci/jeae117 (PMC11288757; doi:10.1093/ehjci/jeae117)

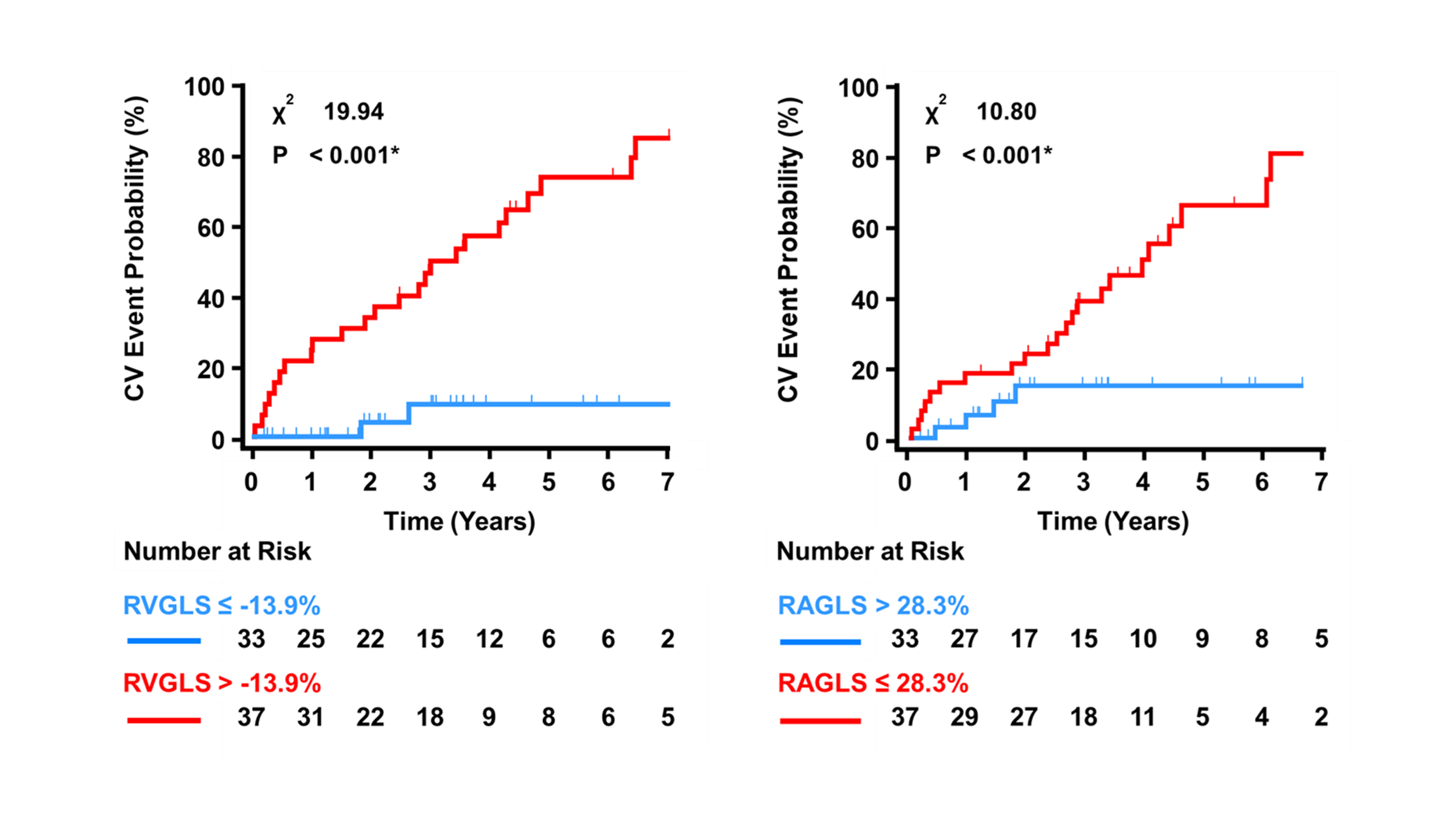

Supplement: jeae117_Supplementary_Data [file jeae117_supplementary_data.zip › Sup F1 RHS ARVC EHJCVI.png]

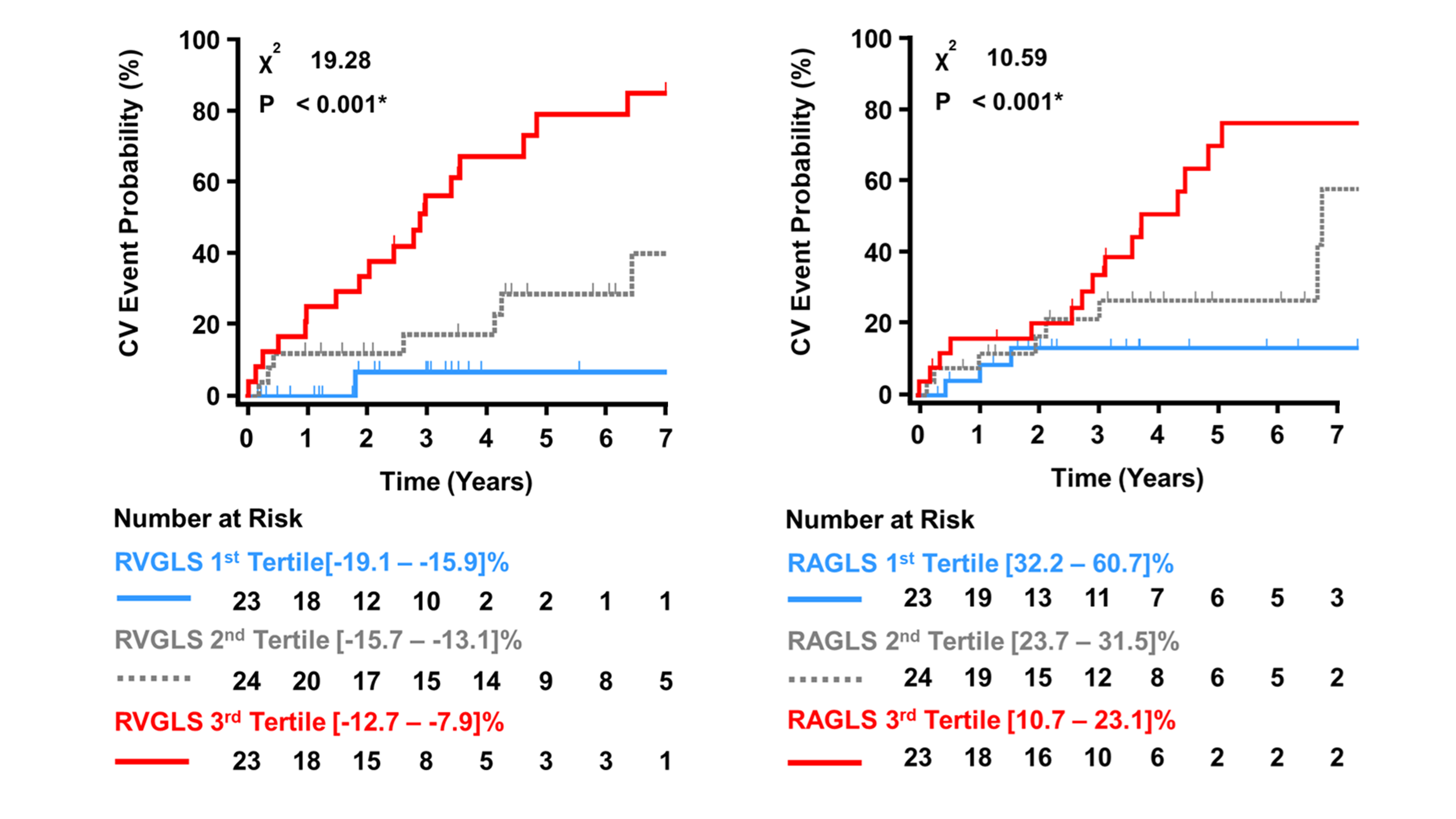

Supplement: jeae117_Supplementary_Data [file jeae117_supplementary_data.zip › Sup F2 RHS ARVC EHJCVI.png]
